# Possibility of a New Indication for Amantadine in the Treatment of Bipolar Depression—Case Series Study

**DOI:** 10.3390/ph13100326

**Published:** 2020-10-21

**Authors:** Marek Krzystanek, Artur Pałasz

**Affiliations:** 1 Clinic of Psychiatric Rehabilitation, Department of Psychiatry and Psychotherapy, Faculty of Medical Sciences, Medical University of Silesia in Katowice, Ziołowa 45/47, 40-635 Katowice, Poland; 2 Department of Histology, Faculty of Medical Sciences, Medical University of Silesia in Katowice, Medyków 18, 40-752 Katowice, Poland; artiassone@gmail.com

**Keywords:** amantadine, bipolar depression, bipolar disorder, off-label treatment

## Abstract

Bipolar disorder is a chronic and remitting mental illness. Antidepressants are not effective in treating acute bipolar depression, and antipsychotic drugs used in the treatment of bipolar depression cause frequent side effects. This situation justifies the search for new drugs as well as the repurposing of drugs used in other indications. In an open and naturalistic serious case study, 4 patients diagnosed with bipolar I disorder, chronically treated with a mood stabilizer, in whom at least two antidepressants were ineffective in the depressive phase, were treated with amantadine. The woman received 100 mg/day and 3 men received the target dose of 200 mg/day. All patients treated with amantadine improved their depressive symptoms after 1 week of treatment. None of them experienced side effects or manic switch. To reduce the risk of a manic switch, the treatment with amantadine was discontinued 2 weeks after the improvement of depressive symptoms, and no recurrence of depressive symptoms was observed. Amantadine may be a further therapeutic option for the treatment of acute bipolar depression. The drug in this indication may act quickly and be well tolerated. Confirmation of the antidepressant efficacy of amantadine in this indication requires replication of the results and conducting clinical trials.

## 1. Introduction

Bipolar disorder type I (BD-I) is a disabling and cyclical mental illness whose essence is mood instability, manifested by recurrent episodes of bipolar depression, mania, and mixed states. In addition to these primary symptoms, BD-I causes chronic symptoms in the form of emotional dysregulation, sleep disturbances, cognitive impairment, and subsyndromal mood disturbances between the episodes [1].

BD-I affects about 2% of the general population; the course of the disease is individual and may differ in severity, duration, and frequency of occurrences [1,2]. Characteristic for BD-I is a longer duration of depressive episodes than of manic episodes. Already Emil Kraepelin described that episodes of depression in bipolar disorder may even persist for years [3]. Contemporary observations indicate an average duration of a depressive episode in BD of 4–5 months, i.e., about 50% longer than the average manic episode [4].

None of the classical mood stabilizers (i.e., lithium, carbamazepine, lamotrigine, and valproate) are well documented for their acute antidepressant effects in bipolar depression [5]. A common practice in the treatment of acute bipolar depression is the use of antidepressants, although they are not effective, and what is more, they increase cycle acceleration, causing a rapid-cycling course, contribute to the formation of mixed episodes and, consequently, reduce the effectiveness of the treatment of bipolar disease [5,6].

In the treatment of bipolar depression, quetiapine, lurasidone, aripiprazole, cariprazine, and a combination of olanzapine with fluoxetine are usually added to the mood stabilizer [5]. It should be emphasized that the use of quetapine, lurasidone, and olanzapine is a frequent cause of the deterioration of the functioning of patients, caused by the frequent occurrence of metabolic, motoric, and cognitive side effects [6].

Other drugs that may be effective in the treatment of acute bipolar depression include *N*-acetylcysteine, vitamins D_3_ or folic acid, hormones tri-iodothyronine or levothyroxine, antibiotic minocycline, anesthetic ketamine, psychostimulant modafinil, and omega-3 fatty acids [6]. All of these adjunctive treatment strategies are off-label indications for bipolar depression, and their efficacy data are mostly derived from single clinical trials, open case series, or case studies.

The limited number of drugs that are effective and well-tolerated in the treatment of a depressive episode in BD indicates the need to seek new drugs as well as repurposing drugs used in other indications. One of the off-label drugs that have an antidepressant effect is amantadine. The molecular mechanism of amantadine’s antidepressant action is not yet fully understood as the drug exerts its pharmacological effects through diverse brain signaling systems such as dopaminergic, noradrenergic, glutamatergic, and opioid systems [7]. Several neurochemical scenarios should be considered; however, the effect of amantadine on dopaminergic transmission is relatively well documented.

**Figure 1:**
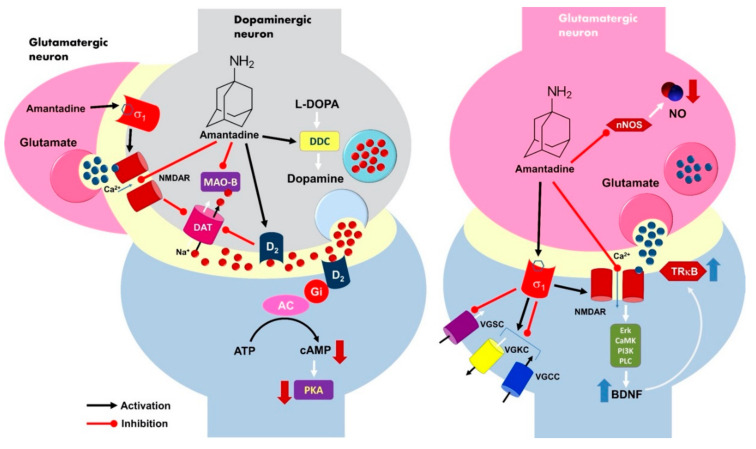
A model of the possible molecular mechanism of amantadine’s antidepressant effects at the level of dopaminergic and glutamatergic transmission. Amantadine indirectly increases the dopamine level within the synaptic cleft due to inhibition of the dopamine transporter DAT1 via the blockage of the presynaptic NMDA receptor ion channel. The activation of the presynaptic dopamine receptor D_2_ may also reduce DAT1 activity in response to the high neurotransmitter level and/or drug binding. D_2_ is a Gi-coupled receptor, and its excitation causes an inhibition of the adenylyl cyclase (AC) activity, decreased cAMP production, and finally, the silencing of the protein kinase A (PKA)-dependent neuronal signaling pathway. Activation of the σ1 receptor can additionally enhance the synaptic dopamine concentration. The σ1 receptor regulates the activity of several voltage-gated sodium, potassium, and calcium channels, G-protein coupled receptors (GPCRs), and some protein kinases. Receptor activation causes a blockade of Na^+^ channels (VGSC), while its interaction with K^+^ (VGKC) and Ca^2+^ (VGCC) channels may be excitatory or inhibitory. The activation of the σ1 receptor results in the enhancement of both NMDA and dopamine D1 receptors. Amantadine is able to inhibit monoaminoxidase B (MAO-B), but it does support L-DOPA decarboxylase (DCC) activity in the presynaptic neuron. The amantadine-related blockade of NMDA receptor function in the glutamatergic neurons causes an upregulation of BDNF and TrkB expression via Erk, CamK, PI3K, and PLC signalling cascade. Amantadine may also inhibit nitric oxide synthase (nNOS) activity and decrease neuronal NO level. All those neurochemical events are considered to be responsible for the generation of the antidepressant effects of amantadine. L-DOPA decarboxylase (DCC); NMDA receptor (NMDAR); phospholipase C (PLC); tyrosine receptor kinase B (TrkB); extracellular signal-regulated kinases (Erk); brain derived neurotrophic factor (BDNF); phosphoinositide 3-kinase (PI3K); calcium/calmodulin-dependent protein kinase (CamK). Up and down thick arrows indicate direction of changes, thin arrows show the course of biochemical reactions, lines with dots indicate functional connections.

Amantadine strongly stimulates dopamine signaling by elevation of the neurotransmitter level within the synaptic cleft via the inhibition of the dopamine transporter (DAT). Dopamine reuptake is regulated with phosphorylation of the DAT1 molecule and probably modulated by presynaptic NMDA receptor activity [8]. Amantadine as a weak noncompetitive NMDAR antagonist accelerates its cationic channel closure, blocking ion current and finally inhibiting receptor function [9]. This follows DAT1 blockage and the increase of local dopamine concentration that plays an important role in the origin of the anti-depression effect [10,11]. Figure 1 presents the entire spectrum of the mechanisms of action of amantadine, proposed and confirmed in the literature [11,12,13,14,15,16,17,18,19,20,21,22,23,24,25,26,27,28,29,30,31,32,33,34].

Clinically, amantadine belongs to the group of dopaminergic drugs increasing dopaminergic transmission, which is considered now as an effective add-on treatment of bipolar depression [6]. The rationale for their use in bipolar depression is a dopaminergic model of bipolar depression pathogenesis, which assumes dysfunction in the brain dopaminergic system of patients with BD [35,36]. Dopaminergic drugs, unlike antidepressants, do not seem to increase the risk of manic switch and are well tolerated by patients [6].

To date, most of the clinical data support the efficacy of amantadine in the treatment of recurrent depression. It was first demonstrated by Vale et al. [37] in a group of 20 depressed subjects treated with amantadine at a dose of 100–200 mg/day, but these results could not be repeated in a study published 2 years later by Rizzo et al. [38]. In the following decades, one clinical trial and several series of cases managed to demonstrate the effectiveness of amantadine in the augmentation of treatment-resistant depression [39,40]. Amantadine was also used in patients with the Borna Disease Virus (BDV), manifesting symptoms of bipolar disorder. In this study, they reported a faster improvement in depressive symptoms compared to patients with major depression symptoms [41]. There are no reports regarding the use of amantadine in the treatment of bipolar depression.

## 2. Results

The addition of amantadine to the mood stabilizer resulted in a significant improvement in depressive symptoms in all 4 patients. In 3 men, the dose of amantadine was increased to 200 mg in the morning due to the lack of improvement in the first week. One week after increasing the dose, the severity of depression in these patients was reduced by 44–57% compared with baseline. In a female patient, after a week of treatment with a dose of 100 mg/day, an improvement of over 66% was observed. Details are presented in Table 1.

The add-on treatment with amanatidine was well-tolerated, and no side effects were observed in any of the patients after adding amantadine. Treatment with amantadine was continued in each patient for 2 weeks after the symptoms improved, and then the drug was discontinued. No manic switch was observed during amantadine treatment, and no recurrence of depressive symptoms after drug discontinuation was observed.

**Figure 2:**
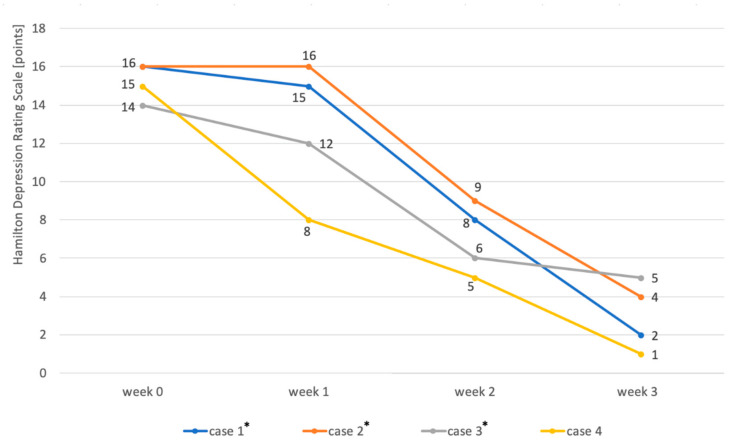
Changes in the severity of depression during the treatment of patients (*n* = 4) diagnosed with bipolar disorder in the depressive phase by adding amantadine to the mood stabilizer. The severity of depression was assessed using the HDRS scale four times—before the start of treatment (week 0), after the week (week 1), after 2 weeks (week 2), and after three weeks of treatment (week 3). The asterisks mark the patients whose dose of amantadine was increased to 200 mg/day in the second week. * the patients whose dose of amantadine was increased to 200 mg/day in the second week.

Individual case reports are summarized below:

1. A 51-year-old Caucasian male diagnosed with bipolar disorder since 2002, when he had his first episode of mania. Since then, the patient was treated with valproic acid in the dose of 1500 mg and lithium carbonate in the dose of 750 mg daily (serum concentration of 0.6 mmol/L). In 2016, he had a moderate episode of depression (16 points on HDRS). The clinical picture was dominated by anhedonia, apathy, lack of energy and motivation, anxiety, and indifference. Further attempts were made to treat depression with quetiapine 300 mg/day, olanzapine 10 mg together with fluoxetine 20 mg daily, and antidepressants: sertraline up to 200 mg daily and escitalopram up to 20 mg daily. However, despite treatment, each time for at least 6 weeks, none of these interventions resulted in improvement. The patient was then offered to add amantadine to lithium carbonate and valproic acid in an initial dose of 100 mg in the morning. After one week, the patient reported a slight improvement (15 points on the HDRS scale). With the patient’s consent, the dose was increased to 200 mg amantadine in the morning. After another week, the severity of depression in HDRS was 8 points. From the patient’s report, the improvement occurred 3–4 days after increasing the dose of amantadine. After another week, HDRS was 2 points (Figure 2). The patient continued treatment with amantadine for another 2 weeks, then reduced the dose to 100 mg in the morning and stopped the drug after three days. During the treatment, no side effects of amantadine were observed, no change in the manic phase, and no recurrence of depressive symptoms after drug discontinuation.
2. A 56-year-old Caucasian male treated for bipolar disorder since 2016, when he experienced a two-month manic episode. He then received valproic acid in a dose of 1500 mg daily and olanzapine in a dose of 20 mg in the evening. His mania symptoms resolved and he was then treated only with valproic acid. In 2017, he reported significant depression (19 points on the HDRS scale). The clinical picture was dominated by tearfulness, sadness, lack of motivation, anhedonia, lack of energy, and difficulty with concentration. He received lamotrigine at the target dose of 150 mg/day, but after 4 months the depression was still there (15 points on the HDRS scale). He then received mirtazapine in a dose of 30 mg in the evening, but after another 2 months, the symptoms remained (16 points on the HDRS scale). The patient was then offered to add amantadine at an initial dose of 100 mg in the morning to valproic acid and lamotrigine. As there was no improvement after one week, the amantadine dose was increased to 200 mg in the morning. After another week, the patient felt better (HDRS 9 points). At the visit after 3 weeks of treatment with amantadine, the patient had an HDRS score of 4 (Figure 2). He continued treatment for another 2 weeks, then lowered the dose to 100 mg in the morning, and stopped taking the drug after 3 days. There were no adverse effects of amantadine and no change from the depressive phase to the manic one, and no relapse of depression after discontinuation of amantadine.
3. A 50-year-old Caucasian man has had bipolar disorder since the age of 31. He started psychiatric treatment in 2013, when the manic episode was followed by an episode of depression that lasted 5 months (12 points on the HDRS scale at the first visit). The patient then received quetiapine, but was unable to function professionally due to sedation at a dose of 100 mg. The treatment was changed to lithium carbonate at a dose of 500 mg per day (serum concentration of 0.6 mmol/L). The patient felt better (6 points on the HDRS scale), but in 2014 there was another episode of depression (14 points on the HDRS scale). The patient then did not respond to 20 mg escitalopram for 6 weeks and then to 150 mg bupropion in the morning for 2 months. He was then offered to add amantadine to the lithium carbonate in an initial dose of 100 mg in the morning. The patient reported a slight improvement at the visit after one week of treatment (12 points on the HDRS scale). Due to incomplete improvement, the dose was increased to 200 mg amantadine in the morning. According to the patient, the improvement was achieved within 2 days (6 points on the HDRS scale after 2 weeks of treatment). After 3 weeks, the HDRS was 5 points (Figure 2). After another 2 weeks, the patient stopped taking amantadine within 3 days. There were no adverse effects of amantadine, no manic switch, and no recurrence of depression after discontinuation of amantadine.
4. A 45-year-old Caucasian woman was diagnosed with BD-I in 2015. She had previously had a four-month manic episode, followed by a depressive episode for three months. She then received quetiapine at a target dose of 300 mg in the evening. The patient tolerated the drug well, but her mood improved only after 6 months of treatment. In 2017, there was another episode of depression (15 points on the HDRS scale), after an earlier short episode of hypomania. She received 30 mg of mirtazapine in the evening, but after 6 weeks there was no improvement and the patient gained 3 kg body weight. She was switched to 150 mg bupropion in the morning, but after another 2 months, there was still no improvement. The patient was then offered to add amantadine 100 mg in the morning to 300 mg of quetiapine. Improvement was observed after 7 days of taking amantadine (HDRS 8 points). After 2 weeks, HDRS was 5 points, and after 3 weeks, 1 point (Figure 2). The patient took 100 mg of amantadine for 2 weeks and then gradually, within 3 days, stopped taking it. As before, no adverse effects of amantadine, no change from the depressive phase to the manic phase, and no recurrence of depressive symptoms after discontinuation of amantadine were observed.

## 3. Discussion

To the best of our knowledge, this is the first study showing the antidepressant effect of amantadine in BD-I. In this case series, amantadine was administered 100 mg in the morning during the first week and was increased to 200 mg from the second week if no clinical improvement was achieved. The adopted dose range and dose building were based on previous studies [37,39,40]. Gradually building up the dose of amantadine appears to be clinically more beneficial than dosing it with a high dose from the start. The probable cause of agitation and aggression observed in 4 people by Rizzo [38], which led to the discontinuation of the study, was the high age of people (68 years and older) and just too high of a dose regime of amantadine (300 mg/day) already at the end of the first week of treatment. Amantadine did not cause side effects in most of the previous studies, as in this study [7].

Amantadine caused a significant improvement in depressive symptoms after the first week of treatment with an effective dose in all the described patients. A similar rate of action of amantadine in patients with BDV with symptoms of bipolar depression meeting the BD-I criteria was reported by Dietrich et al. [41]. Of the 6 patients then treated with BDV infection at a dose of 100–300 mg/day, improvement occurred on average after 2.7 weeks of treatment, although in one case improvement occurred after one week and in two patients after 1.5 weeks. Due to the comorbidity of BDV, these results cannot be fully compared to the cases presented by us.

In the largest clinical trial of additive treatment with amantadine for depression in the course of treatment-resistant unipolar depression, 25 people were treated with amantadine at a dose of 150 mg/day, which was added to imipramine at a dose of 100 mg/day [39]. However, the severity of depression symptoms was assessed only after 3 weeks of treatment. So, it is not known what the effects of using amantadine after 1 and 2 weeks were, although the improvement after 3 weeks was modest, at 11.8% in women and 14.2% in men. In turn, in a study by Stryer et al. [40] in 7 patients with treatment-resistant unipolar depression, the improvement in the severity of depressive symptoms was fast—after 1 week it was as much as 73%. The results of both studies concern the treatment of unipolar depression, in addition to being drug-resistant, and thus may differ from the treatment of bipolar depression. Interestingly, in a study by Stryer et al., the symptomatic improvement was greater and faster in women. Similarly, in the presented case, women suffering from bipolar depression showed improvement faster and after using a lower dose of amantadine.

Of the other drugs used to treat bipolar depression, quetiapine may also cause a rapid onset of its antidepressant effect. It has been shown that compared to a placebo, it begins to be more effective after a week of treatment [5]. This effect is probably related to the increase in BDNF levels in the hippocampus, which is the common mechanism of action of amantadine and quetiapine.

Also, intravenous ketamine given to patients with acute bipolar depression causes a rapid but transient antidepressant effect [42]. The antidepressant effect of ketamine was observed already 2 h after drug administration. The presumably clinical effect of amantadine in depression at doses used in patients is mainly related to its action on NMDA receptors [7]. Since both ketamine and amantadine block NMDA receptors, it can be speculated that it is the blocking of NMDA receptor function, in addition to increasing BDNF concentration, that is associated with the rapid effects of amantadine in bipolar depression.

The treatment of depressive patients with amantadine, despite its clinical efficacy confirmed in studies, is an off-label use, in the sense of repurposing of the drug. The off-label use of drugs is common in psychiatry; in the group of antidepressants alone, off-label indications have been reported in up to 29% of prescribed drugs [43]. Off-label prescribing regulations differ from country to country [44].

**Figure 3:**
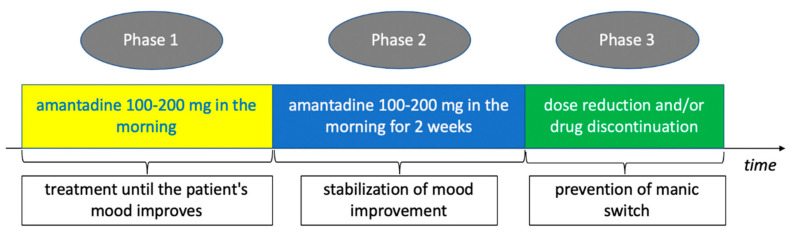
The proposed treatment of bipolar depression with amantadine. The duration of the first phase of treatment (Phase 1) depends on the time when a significant (50%) improvement in the severity of depressive symptoms occurs. Limiting the duration of use of amantadine in the second treatment period (Phase 2 and 3) is due to the minimization of the risk of a manic switch.

The presented case series shows that amantadine is not only effective in the treatment of bipolar depression but also is well-tolerated by patients. Treatment with amantadine for a short, two-week period of stabilizing improvement did not result in cycle acceleration, and discontinuation of the drug after such a period did not cause the recurrence of depressive symptoms. The reason for the short period of stabilization treatment with amantadine was to minimize the risk of manic switch reported in the Letter to the Editor by Sondré et al. [45], in which amantadine was administered to 3 patients with BD-I. Although in the cases described in this report, patients received a high dose of 400 mg/day for 8 weeks, we thought that caution should be exercised in the duration of amantadine treatment after the improvement of depressive symptoms. Our observation is limited by the size of the study group, but it may indicate that in the case of amantadine treatment it is not necessary to use a long period of stabilization of the therapeutic effect. Based on the presented series of cases, a scheme of its use in acute bipolar disorder can be proposed, presented in Figure 3.

The antidepressant efficacy and the observed rapid therapeutic effect of amantadine in a naturalistic open-label study are not yet evidence of amantadine’s efficacy in bipolar disorder, but the results are encouraging and may be the basis for double-blind clinical trials. Confirmation of the efficacy of amantadine in acute bipolar disorder may lead to a new therapeutic indication for amantadine.

## 4. Materials and Methods

The study was an open case series study of outpatients diagnosed with bipolar disorder type I, according to the diagnostic criteria for ICD-10. When the treatment of depression was unsuccessful with at least two drugs used in the treatment of bipolar depression, patients received amanatdine at a dose of 100–200 mg daily. Amantadine was administered only in the depressive phase of at least moderate intensity. The severity of depression was assessed using the Hamilton Depression Rating Scale (HDRS) before taking the first dose of amantadine and then weekly for 3 weeks. Before receiving amantadine, each time the patient was presented with other options of pharmacological treatment and the possibility of inpatient electroconvulsive therapy. None of the patients consented to electroconvulsive treatment. All study patients were receiving at least one mood stabilizer at the time of taking amantadine. In each case, after the end of treatment with amantadine, there was at least a three months’ follow-up for recurrence of depression.

According to legal regulations in Poland, all patients were informed about the mechanism of action of amantadine, its potential side effects, and the off-label use of the drug before starting amantadine. All patients consented to add-on treatment with amantadine.

## 5. Conclusions

Amantadine may be a further therapeutic option for add-on treatment of acute bipolar depression. The drug in this indication may act quickly and be well tolerated. The confirmation of the antidepressant efficacy of amantadine in bipolar depression requires replication of the obtained results and conducting clinical trials.

## Figures and Tables

**Table 1:** Reduction in the severity of depressive symptoms over three consecutive weeks of amantadine treatment. The table shows the doses of amantadine used in the following weeks and the improvement in depressive symptoms as a percentage reduction in relation to the baseline severity of symptoms.

Patients	1-st Week	2-nd Week	3-rd Week
case	daily dose (improvement from baseline)		
1 (male)	100 mg (6%)	200 mg (50%)	200 mg (87.5%)
2 (male)	100 mg (0%)	200 mg (44%)	200 mg (75%)
3 (male)	100 mg (14%)	200 mg (57%)	200 mg (64%)
4 (female)	100 mg (66%)	100 mg (66%)	100 mg (93%)
